# Stakeholders’ Experiences of Research Integrity Support in Universities: A Qualitative Study in Three European Countries

**DOI:** 10.1007/s11948-022-00390-5

**Published:** 2022-08-30

**Authors:** Natalie Evans, Ivan Buljan, Emanuele Valenti, Lex Bouter, Ana Marušić, Raymond de Vries, Guy Widdershoven, the EnTIRE consortium

**Affiliations:** 1 Department of Ethics, Law, and Humanities, Amsterdam Public Health Research Institute, Amsterdam UMC, Vrije Universiteit Amsterdam, De Boelelaan 1117, Amsterdam, Netherlands; 2 Department of Research in Biomedicine in Health, University of Split School of Medicine, Split, Croatia; 3 Population Health Sciences, Bristol Medical School, Bristol, UK; 4 Institute of Clinical Ethics, Francisco Valles, Madrid, Spain; 5 Department of Philosophy, Vrije Universiteit Amsterdam, Amsterdam, The Netherlands; 6 Department of Epidemiology and Data Science, Amsterdam UMC, Vrije Universiteit Amsterdam, Amsterdam, The Netherlands; 7 Center for Bioethics and Social Sciences in Medicine, University of Michigan Medical School, Ann Arbor, USA

**Keywords:** Research quality, Research ethics, Research governance, Research integrity guidelines, Responsible conduct of research

## Abstract

Fostering research integrity (RI) increasingly focuses on normative guidance and supportive measures within institutions. To be successful, the implementation of support should be informed by stakeholders’ experiences of RI support. This study aims to explore experiences of RI support in Dutch, Spanish and Croatian universities. In total, 59 stakeholders (Netherlands n = 25, Spain n = 17, Croatia n = 17) participated in 16 focus groups in three European countries. Global themes on RI support experiences were identified by thematic analysis. Themes identified were: ‘RI governance and institutional implementation’, ‘RI roles and structures’, ‘RI education and supervision’, and ‘Infrastructure, technology and tools supporting daily practice’. Experiences of support differed between countries in relation to: the efforts to translate norms into practice; the extent to which RI oversight was a responsibility of RE structures, or separate RI structures; and the availability of support close to research practice, such as training, responsible supervision, and adequate tools and infrastructure. The study reinforces the importance of a whole institutional approach to RI, embedded within local jurisdictions, rules, and practices. A whole institutional approach puts the emphasis of responsibility on institutions rather than individual researchers. When such an approach is lacking, some stakeholders look for intervention by authorities, such as funders, outside of the university.

**Supplementary Information:**

The online version contains supplementary material available at 10.1007/s11948-022-00390-5.

## Introduction

Problems such as the replication crisis (Ioannidis, 2005; Open Science Collaboration, 2015) and prominent cases of misconduct (Godlee et al., 2011; Kakuk, 2009; Resnik & Shamoo, 2017) raise questions about current research practices and the quality and trustworthiness of research being published. Understandably, concerns about quality and trustworthiness have provided impetus for normative guidance (Anderson, 2014; Aubert Bonn et al., 2017; Godecharle et al., 2014; Resnik et al., 2015) and for practical measures to support good research conduct (Kalichman, 2014; Marusic et al., 2016). Practical measures typically target individual, institutional, or broader structural levels—for example individuals are encouraged to follow reporting guidelines (Simera et al., 2010), institutions are incentivized to provide Research Integrity (RI) training (Marusic et al., 2016), and, on the structural level, policymakers are calling for open science and an overhaul of the researcher evaluation process (European Commission, 2015; Moher et al., 2020). Evidence of the effectiveness of these different interventions—alone or in conjunction—is lacking or of poor quality (Marusic et al., 2016, Tijdink et al., 2021). There is, however, a general consensus that multilevel action is needed to improve RI (Forsberg et al., 2018; LERU, 2020; Mejlgaard et al., 2020). This paper focuses on support for RI in European universities. The European Code of Conduct for Research Integrity (ALLEA code) states that institutions are responsible for: promoting a good research culture, responsible policies and procedures, proper infrastructure, training, adequate data management, and proper handling of suspected violations of research integrity (ESF-ALLEA, 2017). For this study, we start with the assumption that support should be sensitive and responsive to stakeholders’ actual experiences of interventions, and that a qualitative exploration of these can reveal important insights into the perceived sufficiency or otherwise of interventions in practice.

A number of qualitative studies have already provided some nuanced insights into how measures to improve RI are experienced in practice. Previous research has revealed researchers’ low awareness of RI guidance, or hostility towards guidance that overburdens or places too much responsibility on researchers (Davies, 2019; de Vries et al., 2006), and support for measures that acknowledge that the research community as a whole is responsible for RI (Hyytinen & Löfström, 2017). The negative influence of extreme power differentials and production focused assessment procedures on RI (de Vries et al., 2006; Geller et al., 2010; Haven et al., 2020) and the influence of country and institutional culture on research practices (Haven et al., 2020; Olesen et al., 2017) have also been reported. Opaque procedures and fears of retaliations have been identified as barriers for researchers to report misconduct (Godecharle et al., 2018; Mecca et al., 2014; Satalkar & Shaw, 2018). Researchers have also been reported to have a preference for constructivist and discipline sensitive approaches to RI teaching (Hyytinen & Löfström, 2017; Sarauw et al., 2019), and to stress the importance of responsible mentoring and supervision for good research conduct (Haven et al., 2020; Hyytinen & Löfström, 2017; Olesen et al., 2017).

There are, however, few cross-country studies. Cross-country studies can foster a more broadly informed perspective, highlighting similarities between countries and good practices that could be implemented elsewhere, as well as drawing attention to issues unique to specific countries. In Europe, such a perspective is particularly important to inform European level developments such as the European Code of Conduct for Research Integrity (ESF-ALLEA, 2017), and proposals for official European bodies to investigate allegations of breaching RI (Bendiscioli & Garfinkel, 2020). Cross-country studies on RI support have primarily focused on comparing policies and oversight structures rather than exploring stakeholders’ direct experiences (Aubert Bonn et al., 2017; Godecharle et al., 2014; Marusic, 2019). It is particularly important that cross-country studies compare the experience of support from the perspective of the study participants because RI support may look different in different countries—support might go under a different name or be provided via different roles or structures.

Our study takes a cross-country approach to better understand the experiences of RI support in Europe. Here, we understand RI as referring to “the principles and standards that have the purpose to ensure validity and trustworthiness of research” (WCRI, 2020) in contrast to the closely related area of research ethics (RE) which concerns “the moral problems associated with or that arise in the course of pursuing research” (Steneck, 2006). The research question which guides this paper is: what are the experiences of RI support of people involved in the research process in Dutch, Spanish and Croatian universities?

## Methods

The study draws on data collected as part of a broader stakeholder consultation conducted for the EnTIRE project, a European Funded project that builds an online platform for researchers (www.embassy.science) which explored the RI and RE support experiences in three European countries at diverse levels of research and innovation development (The Netherlands, Spain and Croatia) (European Commission, 2017). The consultation used a qualitative approach, consisting of focus groups with stakeholders involved in the research process. The study protocol is available at https://osf.io/tf8mc/. In this paper, we report the findings related specifically to experiences of RI support in universities.

### Country Selection and Recruitment

The Netherlands, Spain and Croatia were selected to represent European countries that have national laws, bodies, and codes governing RI, but which are diverse in terms of research and innovation activities (European Commission, 2017), geographical location, language and culture. This approach maximises diversity within a common normative environment as specified in the European Code of Conduct for Research Integrity (ESF-ALLEA, 2017). Practically, the authors also had access to experts in these three countries to offer essential contextual expertise. Major stakeholders (Table 1) in the research process were purposively recruited to the focus groups using both general and targeted recruitment strategies. Because the general aim of the stakeholder consultation was broader than the scope of this paper, some stakeholders outside of unviersities were targeted, however, due to the nature of research careers and collaborations, almost all stakeholders had some role within a university or closely collaborated with stakeholders from universities. General strategies included circulating the call on social media (Linkedin and Twitter) and advertising it on the EnTIRE project webpage, whereas targeted strategies are described in Table 1. Before the focus groups, participants received, via email, an information letter, and some brief questions on their personal characteristics. Participants signed informed consent and confidentiality agreements.

**Table 1:** Targeted recruitment strategies per stakeholder group

Stakeholder	Targeted
Researchers (various disciplines)	Advert sent to 42 discipline specific learned societies professional societies (list obtained ISE, 2017)
	Advert circulated on general mailing lists by contacts in universities local to the focus groups
	Specific researchers invited from local universities
Journal editors	Advert published on World Association of Medical Editors mailing list and a closed BMC editors LinkedIn group
RE or RI committee members	Advert circulated amongst ENERI and ENRIO member
	Members of the Committee on Publication Ethics (COPE) from the three countries were directly approached
Research managers	Specific research managers invited from local universities
Policy-makers	Specific institutional policy makers invited from local universities
	Invites sent to relevant national policy making organizations
Industry representatives	Advert sent to relevant in-country research intensive companies
Research funding agency	Advert sent to relevant in-country national funding organizations

### Data Collection

**Fig. 1:**
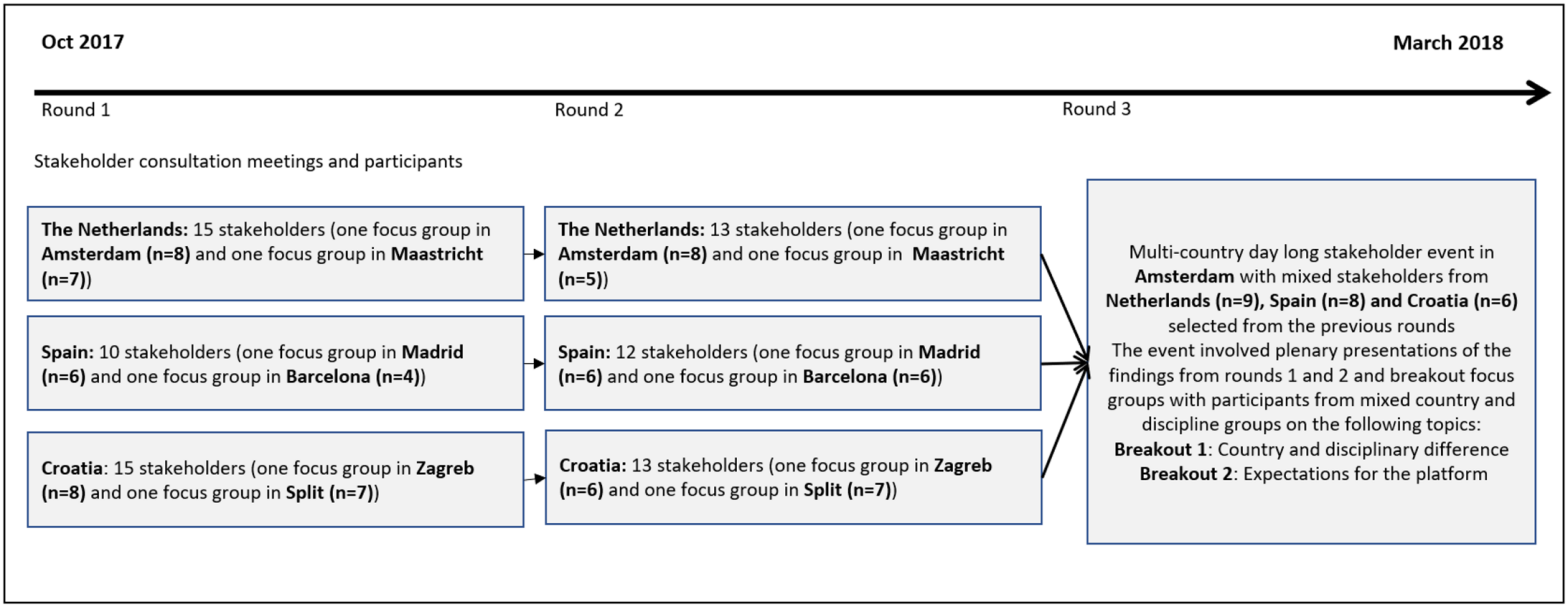
Stakeholder consultation meetings and timeline

The consultation aimed to recruit a group of stakeholders from each of the three countries to participate in three consultation rounds between Oct 2017 and Feb 2018. The first two rounds were conducted in stakeholders’ own countries, whereas the final round was held in Amsterdam with a selection of participants from each of the three countries. Whilst it was envisoned that the same participants would participate throughout all rounds (Fig. 1), in practice there were some drop outs and new recruits during the consultation. Efforts to recruit new participants were focused on inviting PhD students, who were particularly under-represented in round 1 (Table 2).

**Table 2:** Participant characteristics for each individual round and across the whole consultation

Participant characteristics	Consultation round			Across all arounds ^a^
	Round 1	Round 2	Round 3	
	n = 40	n = 38	n = 73	
	n (%)	n (%)	n (%)	n (%)
Country				
The Netherlands (NL)	15 (38)	13 (35)	9 (40)	25 (43)
Spain (ES)	10 (25)	12 (32)	8 (35)	17 (29)
Croatia (HR)	15 (38)	13 (35)	6 (27)	17 (29)
Age				
20–29	3 (9)	6 (21)	3 (16)	6 (13)
30–39	13 (41)	10 (34)	8 (42)	18 (39)
40–49	12 (38)	8 (28)	6 (32)	14 (30)
50–59	3 (9)	4 (14)	2 (11)	5 (11)
60–69	1 (3)	1 (3)	0 (0)	3 (7)
Gender				
Female	22 (55)	24 (64)	12 (53)	26 (45)
Role				
Researcher	25 (63)	26 (69)	19 (83)	40 (68)
Member of a research ethics or research integrity committee	14 (35)	8 (22)	7 (31)	18 (31)
Policy maker	6 (15)	2 (6)	2 (9)	6 (11)
Research manager or administrator	7 (18)	5 (14)	2 (9)	9 (16)
Journal editor or assistant editor	7 (18)	7 (19)	3 (14)	11 (19)
Working for a research funding organisation	3 (8)	4 (11)	1 (5)	4 (7)
Research policy, training, or compliance officer in industry	1 (3)	0 (0)	1 (5)	2 (4)
Other	3 (8)	3 (8)	2 (9)	4 (7)
Highest departmental research position (if applicable)^c^				
Ph.D. Student	3 (8)	7 (19)	6 (27)	9 (16)
Mid-career (Post doc, senior researcher, assist. or assoc. prof)	14 (35)	14 (37)	9 (40)	20 (34)
Established researchers (Prof. and Heads of Dept.)	7 (18)	3 (8)	4 (18)	8 (14)
Missing*	1 (3)	2 (6)	0 (0)	3 (6)

^a^The sum of the roles represented exceeds the number of participants because participants could select multiple roles

^b^The representation of different stakeholder groups over all participants (n = 59) is not the sum of rounds 1 to 3 because many stakeholders participated in multiple rounds

^*^Number of participants who identified as researchers who did not indicate a departmental position

The first consultation round focused on a broad exploration of how RI and RE are understood and supported in institutions. The second round centered on participants’ preferences for online RI and RE support to be developed by the research project that funded the stakeholder consultation (EnTIRE), and the third round brought together stakeholders from different countries to inform them about the preliminary findings from each of the three countries and to jointly discuss these findings in breakout room focus groups on (1) understandings of RI and RE (two groups) and on (2) differences in experiences and needs between countries and disciplines (two groups). In rounds two and three the results of the round before were presented and discussed with participants to both gauge the descriptive accuracy of the emerging themes and to allow new participants to share their experiences related to the topics of the previous round. Topic guides, consisting of open questions and possible prompts, were developed for each round (see Table S1) by a multidisiciplinary team (representing social science, ethics, psychology, and online design). Focus groups were moderated by experienced qualitative researchers (NE and GW) and reporters (CV, EV, IV, GW, AM, HD or JL depending on location). The main interviewer (NE) was in contact with participants by email before the consultation, presented herself as a fellow researcher (postdoc), and emphasized that the participants themselves were the experts. Furthermore, although having worked in research in the Netherlands and Spain, the main interviewer – as a Brit – retained an ‘outsider’ status and perspective. The discussions were conducted in local universities or private facilities at two geographical locations in each country (See Fig. 1) to improve in-country accessibility. Round 1 and 2 focus groups lasted approximately two hours, whereas round 3 focus groups lasted approximately one hour. All discussions were in English and digitally recorded.

### Analysis

Recordings were transcribed verbatim by professional transcribers under a confidentiality agreement, and subsequently checked for quality (NE, ND, and IB) before deletion. Anonymized data were analysed thematically using a combined deductive and inductive approach (Fereday & Muir-Cochrane, 2006). All data analysis was conducted in R 3.4.4 Qualitative Data Analysis package (https://rqda.r-forge.r-project.org/). Initial, deductive, codes were inspired by (1) institutional responsibilities outlined in the ALLEA code, and (2) the categories of data provisionally being collected for the project’s online platform. These codes included ‘Codes, guidelines, and standards’, ‘Legislation’, ‘Policies’, ‘Committees’, ‘Education’, ‘Expert advice and contacts’, ‘Cases, casuistry, and scenarios’. The deductive approach offered preliminary support categories to begin a line-by-line coding independently by two researchers (NE and IB), during which new codes (e.g. ‘technological innovations to support RI’ and ‘audits’) and sub-codes (e.g. ‘content’, ‘delivery’ and ‘cases’ within ‘education’) were inductively developed, compared, and discussed within the research team. The inductive approach allowed for the identification of support that participants themselves found useful as well as more specific institutional approaches (e.g. audits). Preliminary analyses between rounds enabled the interviewer to ask about sources of support known to the research team and identified by participants across the three countries. Codes were iteratively refined and memos were kept to clarify code meanings and code inter-relations. In this paper, we present only data related to participants’ RI support experiences. Participants had diverse understandings of RI, therefore we operationalise the term as referring to “the principles and standards that have the purpose to ensure validity and trustworthiness of research” (WCRI, 2020). Codes related to RI support experiences were organised into potential themes which were reviewed and refined by the research team to minimize overlap and ensure the best descriptive fit to the data. In analysing themes, particular attention was paid, when relevant, to differences between countries, disciplines, and to divergent experiences and opinions. Information saturation was considered to have occurred when no new codes were identified and the information on identified codes was sufficiently dense. The final coding scheme is provided in Table S2. A final re-reading of transcripts, after identifying the final themes, allowed for a recontextualization of the cross-cutting themes for each country which is reflected in the theme descriptions (Ayres et al., 2003). Approaches taken to ensure the trustworthiness of the qualitative analysis included independent coding by two researchers to foster dialogue and agreement on data interpretation within the research team, transparent reporting (following the SRQR checklist) and conducting a ‘member check’ after each focus group round (Braun & Clarke, 2013).

## Results

### Participants

In total, 59 individuals participated in the consultation (Netherlands n = 25, Spain n = 17, and Croatia n = 17), with many participating in multiple rounds. Figure 1 illustrates the location and number of participants per round. As mentioned above, whilst it was hoped that the same participants would join all rounds, there were some drop outs and, subsequently, new recruits were purposively selected (16 to round 2, 3 to round 3). Table 2 shows the participant characteristics per consultation round and across the consultation as a whole (for individual country breakdown, see supplementary Table S3). Participation was greatest from the Netherlands (43%), followed by Spain and Croatia (both 29%). The age category most frequently represented was those aged 30–39 (39%), and gender representation was fairly even (45% female). Almost all stakeholders had multiple roles and represented more than one stakeholder group. On aggregate, however, the largest group represented was researchers, followed by members of RE or RI committees, journal editors or assistant editors, research managers, policy makers, funders, and representatives from industry (Table 2). Researchers from different career stages were represented: 16% of participants were early career researchers (PhDs), 34% were mid-career (Post docs, Assistant and Associate Professors) and 14% were senior researchers (Professors and Department Heads).

### Thematic Findings

The final themes represent the different levels at which RI support was experienced by participants in the three countries:

1. RI governance and institutional implementation
2. RI roles and structures
3. RI education and supervision
4. Infrastructure, technology and tools supporting daily practice

These themes are described below, with selected illustrative quotes in the text and additional illustrative quotes for each theme from each country provided in supplementary Table S2.

#### RI Governance and Institutional Implementation

The Netherlands, Spain and Croatia have national guidance and structures governing RI and are similarly subject to broader European codes and regulations, such as the Revised European Code of Conduct for RI (ESF-ALLEA, 2017) and the mandatory General Data Protection Regulation (GDPR) (https://gdpr-info.eu/). European level guidance was described as providing the impetus for positive changes in national guidance, particularly in Croatia and Spain, even if implementation within existing regulatory systems could require a degree of adaptation to specific contexts. There were however differences between countries in how participants discussed national RI codes. Diverse Dutch stakeholders, for instance, referred to the Netherlands Code of Conduct for RI (2004, updated 2018)(VSNU, 2018) with some anticipating the update of the code and the implications for their institution codes and practices.

[W]hat we do with the policy advisers of the academic medical centers is that we get the concept code now, and we see, well, what's different from the old one. And how does it affect the policies and codes we have in our own medical academic center, so it could be that the research code we have is going to be changed because of the new code.

P4, Female, Policy-maker, the Netherlands

In Croatia, participants discussed the Ethical Code of the Board of Ethics in Science and Higher Education (2006, updated 2015) (Agency for Science and Higher Education, 2015), however it was described as being adequately formulated but not sufficiently adhered to in research practice, often due to external social pressures.

[W]e have a code of conduct which was established in 2006, basically, so in Croatia it isn't a problem to have everything set up on the place, it's a problem with how this is working in reality. So that is the problem. When you have corruption, when you have politics which is going inside RI, inside the science system, and know that this is a problem in Croatia.

P34, Male, Researcher and RE or RI Committee Member, Croatia

Spanish participants discussed European guidance and national laws; however, the Spanish National Statement on Scientific Integrity (COSCE-CRUE-CSIC, 2015) was not mentioned at all*.* Participants from all countries described some researchers as lacking awareness of institutional RI codes however, and expressed frustrations at insufficient efforts to translate guidance into practice; indicating that, in the absence of commitment to implementation, institutional level RI codes and policies can actually create dissatisfaction. Participants described a need for sustained commitment from the highest ranks of the university, action plans for the implementation of guidance in research practice, and designated persons responsible for overseeing that implementation (e.g., research managers, research funding officers, RI officers).

You need the directors of the centers, or the manager or whatever, to actually be interested in this and say and “I'm going to, every year when I do the annual talk to my whole center, I'm going to touch on the issue of RI, and gender” [which was discussed as another cross-cutting issue], just to show that it is important.

P18, Female, Research Manager, Spain

Some participants called for more far-reaching regulation of the research process. A few Croatian participants even called for EU regulation enforcing mandatory research audits. Others however argued that, ideally, science should be self-regulating, and rejected the additional bureaucratic burden that comes with greater oversight.

#### RI Roles and Structures

Participants frequently discussed the roles and structures put in place in institutions to support RI. Important sub-themes were: (1) People with designated RI support roles and (2) Committees Supporting RI.

##### People with Designated RI Support Roles

In the Netherlands, many participants described well-defined structures and roles, including RE committees (for ethical aspects), scientific committees (for scientific quality), clinical research offices (for safety and quality of trials), RI committees (for misconduct allegations), RI counsellors (for confidential advice), quality commission (for random audits) and other experts (e.g. data protection officers, library staff, and RI teachers). Although not all roles and structures were available in all participants’ institutions, and the availability of support could differ depending on discipline and if research was required, by law, to pass through an accredited RE committee (i.e. interventions on animals or humans/personal data) or not. Despite quite clearly delineated roles and responsibilities, problems could however arise if advice was not consistent between different sources. For example, one researcher discussed her frustrations at the lack of consistency in the advice on data management given by the RE committee, the data protection officer, and the clinical research office.

We had a trial, and it was finished, so then of course, you let all the institutions know that you are finished, then you get information back what you should do now. So, the privacy officer, he said 'well, you should immediately destroy the connection between your persons and data. You should immediately destroy the connection.' And that surprised us. So, then we called our monitor from the Clinical Research Bureau, and they said, 'oh, we don't have an opinion, just do what the privacy officer says.' And then we thought, 'well, what would the medical ethical [RE] committee have to say?' And they said, 'no, you should save it for five years.' So then, yeah, well, what do you do?

P2, Female, Researcher, the Netherlands

The accessibility and availability of other experts for RI support was less elaborate in the other countries. In Spain, participants described research funding officers and research methodology advisors of the Research Unit and the Quality Unit as important sources of support. Croatian participants, in contrast, described particular difficulties finding confidential and independent RI advice during a project and often turned to more senior colleagues for advice. Croatian participants’ also experienced difficulties getting RI advice from a RE committee during a project, which is described further below.

##### Committees Supporting RI

Perhaps counterintuitively for some, RE committees were often discussed in relation to RI support; particularly in Spain and Croatia where they are responsible for promoting RI and dealing with research misconduct cases and also in the Netherlands for certain types of research.

In the Netherlands, participants from law, computer science, engineering and social sciences described the development of non-accredited ethics advisory committees at departmental, faculty or institutional level to provide advice on research not requiring ethical review by law, since no interventions on human subjects are involved. The advice of these committees has no legal status; their aim is to improve the quality of research and to strengthen departmental, faculty or institutional responsibility. In these committees, there was little separation in the organisation of RE and RI support, either on the level of the responsible committee or the people involved in providing advice. For example, a member of the faculty of law described the combined integrity and ethics roles of their advisory committee because they “were unable to split it up” (***P43***). Challenges for non-accredited committees included a lack of legal status of their advice, and the small volume of cases which created difficulties for committee members to develop expertise in providing advice.

In Spain, RE committees are responsible for the promotion of RI and in dealing with research misconduct cases. Furthermore, participants described accredited RE committees increasingly following-up on research after authorization. One RE committee member described auditing a project two years after study approval on research outcomes and financial issues.

P54: Assessing what was happening after our approval, we have learnt that the investigators, the researchers, forgot all the things that they are going to do […] it's one of the most important things for us that they have planned it well, but they are [also] doing well and they are publishing well.

I: So, you actually follow them through the whole process?

P54: We have begun two months ago only.

I: Okay, and what is your experience of that?

P54: Our experience is that like a wall, big high wall, they are, they have to learn because they have to explain that this public money has been well invested.

P54, Female, RE or RI Committee Member, Spain

In this example, the participant refers to research for which post authorization follow-up is not obligatory but “less than an order, but more than a suggestion” (***P54***) because the same committee would appraise protocols from the researchers in the future and because the committee tends to apply the same procedures for studies that are legally required to submit to a post authorisation audit (namely those involving pharmaceuticals or observation studies in healthcare settings) to the majority of research protocols approved.

In Croatia, RI is not differentiated from RE (indeed RI is difficult to translate in Croatian), and RI is considered a part of RE. RE structures, therefore, also serve RI functions, and RE committee members described being asked to give opinions on allegations of misconduct. However, due to a lack of authority and, sometimes, expertise, RE committee members expressed reluctance to do so. RE committee members also described having little power in such cases: their advice may or may not be followed by a faculty Dean.

P28: For instance, there was a case where some results were published in a bad journal and then sent to a good journal and it was claimed that the number of data was much much bigger, but the curves are completely the same. You cannot have the same average and the same statistical error; it was 10 times or 100 times bigger number of data. This was 100% proof that this was wrong. Nevertheless, other members of the committee did not want to prosecute or to make any strong decision, and the complaint came from the colleagues.

P28, Male, Researcher and RE or RI Committee Member, Croatia

Croatian researchers were deterred from seeking RI advice from a RE committee, however, because the committee must start a formal process if a person’s name is mentioned in relation to questionable research practices. This requirement was unique to Croatia, however participants from all countries described difficulties in seeking advice from RE committees after approvals were granted.

In Croatia and Spain, there are no formal RI committees. As mentioned above, RE committees deal with RI complaints. In contrast, in the Netherlands, RI committees deal solely with misconduct complaints. In all countries, the advice offered by committees in relation to RI breaches carries little legal authority. Some participants described concerns about the credibility of RI-related expertise of some members nominated to institutional or national committees and a need for education and support of their members.

Actually, it's my personal opinion about how we're dealing with this RI is quite critical to how we're dealing with cases of research misconduct and one of the reasons is that you let these peers deal with these cases but they have no actual skills to do that, they're just professors who voluntarily, or sort of less voluntarily, applied to do it and who do it on top of their job without any training, without any guidance, without any support actually.

P10, Female, RE or RI Committee Member, and Policy Maker, The Netherlands

Croatian participants described concerns about external pressures on RI investigations, and referred frequently to the Croatian parliament’s attempts to limit the remit of the National Committee for Ethics—which deals with alleged cases of misconduct—because it ruled unfavourably in the case of a former science minister accused of plagiarizing a part of his doctoral thesis (See **P34** quote under the first theme). These pressures were not, in contrast, reported by participants from Spain or the Netherlands, or in the multi-country focus groups where the Croatian situation was discussed in some depth.

### RI Education and Supervision

Participants from the three countries frequently emphasized the need for education to create RI awareness, adherence to RI in practice, and internalisation of RI ideals. Dutch participants however described more experiences with formal RI education, with some describing RI training as an obligatory part of PhD education and even, according to one participant, embedded in their university’s competency model.

At my university we made research integrity a learning line in our competence model, so every course has to think about what issues of research integrity do we want to address to our PhD students.

P10, Female, RE or RI Committee Member and Policy-maker, the Netherlands

In Spain and Croatia, some pioneer initiatives were described, which had received increasing interest from other institutions, sometimes in response to the demands of funders. These, however, were often voluntary and included in only specific courses. In all countries, RI education was described as being predominately targeted at PhD students, with the training of more senior researchers considered important but difficult to enforce. Due to senior researchers’ perceived aversion to RI training, some participants suggested making the training mandatory or integrating it into other continuous education courses. A few also suggested that RI education should be offered to all members of an institution’s staff, including project managers, IT support, and clinicians conducting research in academic hospitals, to promote an organisation-wide culture change.

In regard to the content of courses, Croatian participants expressed a greater need for training materials, more frequently referred to RI training as narrowly concerning ‘falsification, fabrication and plagiarism’, and criticised Croatian approaches compared to what they perceived as more ‘positive’ approaches in other countries.

What is typical in Croatia, is that we find a lot of regulations, and lot of consequences, so 'if you will plagiarize, then you will, you know, something will fall from the sky and kill you.' But actually, nothing is happening, and there are no actually educational materials, there are not guidelines, anything, but just some kind of rules and threats, that's all what we find. And we compared it to international university, and you can see almost immediately how they are trying really to educate, not to blame someone.

P36, Female, Researcher, RE or RI Committee Member, Croatia

In contrast, Dutch and Spanish participants who delivered RI training described using interactive approaches based on real cases of questionable research conduct that resonate with researchers’ day-to-day dilemmas.

Participants often, however, emphasized that education alone would not improve research practices, but needed to be implemented in conjunction with other measures. The emphasis on which other measures were most complementary depended very much on each participant’s personal preference; ranging from calls for multiple levels of support, to a focus on specific interventions (e.g. protocols close to research practice). One participant linked this back to institutions taking responsibility for RI rather than shifting that responsibility onto researchers.

We tend to put the blame on the individual and take it a long way from the responsibilities of universities. And I think when you approach them as, when you teach research integrity, ideally you want the right resources to prevent this at the university/institute level, that could prevent data manipulation, that could have to do with data storage.

P35, Male, Researcher, Croatia

A few participants stressed the need for more reflexive research practice, however, found it difficult to describe how that might be achieved as it would require a change in research culture rather than training.

An informal aspect of RI education that was frequently discussed was learning about research from more senior colleagues. Indeed, participants from all countries often described senior researchers as the most important influence on their own research practice. Although some Dutch participants described departmental initiatives to promote good role modelling and develop coaching skills, participants from all countries also frequently questioned the knowledge and behaviour of senior researchers and, as mentioned above, very few participants described obligatory RI training for more senior researchers.

In these sessions, senior, but also the juniors, are stimulated to share like, for instance, if you published an article, and then in hindsight you realized 'oh my god, I did one of the analyses in the wrong way, what should I do?' So, we really want to stimulate that the juniors are not keeping it to themselves, but share them, and then we can cope with it all together.

P2, Female, Researcher, the Netherlands

### Infrastructure, Technology and Tools Supporting Daily Practice

Participants’ discussion of infrastructure, technology and tools focused on their potential to simplify and guide researchers through complex processes and/or to prevent questionable research practices through greater transparency. Because the focus groups took place around the 2018 reform of EU data protection laws, participants from all countries frequently discussed data management and often described a lack of adequate infrastructure, tools, and practical guidance on data storage and transfer as hindering compliance with the new law.

[w]e're struggling more with the infrastructure part. I don't know if it's in your organisation for a researcher to find his way to get advice on data management or advice on storage or computers or whatever, well that's really a challenge for us at this moment. And that's not only on data infrastructure but that's a big part of it. So that's maybe the support we're missing.

P15, Female, Policy Maker and Other, The Netherlands

Across the three countries, participants commented on diverse levels of awareness within teams and between disciplines. The diversity of data handled and difficulties understanding regulations and good practices related to specific types of data was a key challenge. Indeed, differences between disciplines were more noteworthy than differences between the three countries, a distinction which was also commented on directly by one participant working on a European project:

We were very aware about going through an ethics committee, about writing protocols, about data management. Comparing with other stakeholders who were maybe from countries that we, from a prejudice let's say, we could think they were more strict, from Northern Europe. And they were less because they were from another field […] and they were not so used to working with participants on things like that.

P50, Female, Researcher, Spain

Although participants overwhelmingly focused on data management when discussing infrastructure, technology and tools to support research practice, some other supportive tools were described. For instance, Open Science platforms for making data and code available, software to check for plagiarism or to check analyses, and authorship agreements. Some concerns were raised that such tools might be inadequate to solve complex RI problems. Authorship agreements, for example, were considered difficult to negotiate or enforce considering the hierarchical structures within teams. Open Science platforms were also criticised because, although data and code are more readily available, they are still rarely checked.

So, I think the first step is everything should be available, but the second step should be we should also check. I mean, this is sort of the premise of science, right? We check things by other people and then we say it's okay, then it's published. That’s kind of the premise, and we're not doing that with data, with programmes, with things. We say they're available, we've read the paper.

P13, Male, Researcher, RE or RI Committee Member, The Netherlands

## Discussion

This study of RI support-related experiences across three European countries revealed a number of differences between countries, notably, in the proactiveness of institutional RI governance and oversight (e.g. if policies are supported with action plans for implementation, or if oversight involves research audits), and the extent to which RI oversight was considered a responsibility of RE structures, or separate RI structures. Differences were also described in the availability and quality of support activities close to research practice, such as tools, training, responsible supervision, and having a credible and accessible person to go to for confidential advice.

### RI Governance and Institutional Implementation

The differences in perceived effectiveness of, or adherence to, normative guidance between countries with similar availability of national codes or statements on RI, provides additional information compared to cross-country analyses that look solely at policy differences between countries or institutions (e.g. Anderson, 2014; Aubert Bonn et al., 2017; Godecharle et al., 2014; Resnik et al., 2015). These differences in experiences highlight the need to pay attention to the social, cultural, and political context in which a policy or other support measure is implemented. For example, the outside pressures influencing RI experienced by Croatian participants have been recognised as challenges before (Foltýnek & Dlabolová, 2020), indicating that additional attention to implementation might be needed when such barriers are persistently identified. They also highlight the added value of approaches that look at the influence of these guidance documents at an institutional level in practice and teaching (Davies, 2019; Sarauw et al., 2019).

Participants’ experiences in practice reveal that normative guidance is vital, but so are support measures to facilitate its enactment in practice. Participants from the Netherlands and Spain highlighted the need for sustained commitment to the implementation of RI norms within institutions at the highest levels of the university, implementation of action plans, and designated persons responsible for the translation of guidance in practice. Although the job title of these ‘responsible persons’ differed between countries, and even institutions, key characteristics included accessibility and the ability to act as an intermediary between policy and practice. This need for a role between policy and practice has been emphasized previously in policy statements related to institutional responsibility (Forsberg et al., 2018; LERU, 2020) and has been raised specifically for Croatia (Buljan et al., 2018). Indeed, since the end of data collection, one Croatian institution has introduced the position of an RI advisor (MEFST, 2020). Without this attention to implementation and translation of normative guidance into practice, guidance runs the risk of protecting institutions but failing researchers.

### RI Roles and Structures

The study also revealed differences between countries and disciplines in the role of *RE* committees for *RI* oversight. These findings reflect differences between countries and disciplines in the degree to which RI is considered part of RE and in the legal criteria related to the assessment of proposals or allegations. A diversity of other positions with RI responsibilities were also described, with little overlap between countries. Furthermore, experiences of, or desires for, ‘research audits’ were described—with the responsible party (actual or desired) differing between countries. In the Netherlands, audits were conducted randomly in some institutions by a quality commission. In Spain, all observational studies conducted in the health care service and all research involving pharmaceuticals is required to submit to post authorisation audit by RE committees (Spanish Ministry of Health and Social Services, 2009; Spanish Royal Decree, 2015). Spanish RE committees sometimes apply the procedures to all research protocols, even those for which post authorisation audit is not mandatory, resulting in more and more research being subject to post authorisation follow-up. In Croatia, audits were not conducted, however some participants called for them and considered funders as a potential independent party that could carry out such checks. The need to check open data and code, including automated checking, was also touched on.

Although there have long been calls for retrospective review by RE committees or for research data audits to improve accountability in research (Click, 1989; Dawson et al., 2019; Shamoo, 2013), such retrospective review is still rare. The initiatives in the Netherlands and Spain can offer inspiration about how to embed such processes in existing oversight structures. Furthermore, the jurisdictional differences between countries revealed in our study, where the responsibility for different aspects of RI issues might fall to different committees and positions depending on the country or discipline, raise questions about the appropriateness, or feasibility of, European-level guidance that recommend specific separate RI structures or roles within institutions (Forsberg et al., 2018; LERU, 2020) and might rather support the translation of oversight responsibilities into local jurisdictions, rules, and practices. The development or desire for audit procedures by different structures, each with unique credibility and authority within a specific country context, is a case in point.

### RI Education and Supervision

Our study also highlights the importance of education and responsible supervision to translate RI guidance into practice. However, despite recommendations in the European Code of Conduct for Research Integrity for research institutions to provide RI training for all researchers across the career path, provision of RI education was described as piecemeal, often voluntary, and mostly lacking for senior researchers. Considering that senior researchers are often the first source of RI advice and have an important modelling role (Anderson et al., 2007), a lack of targeted initiatives and mandatory education for this group potentially limits the impact of other RI measures. The importance of RI education not only for PhDs, but also senior researchers and other research staff, such as lab technicians and research managers, supports recommendations from previous studies (Godecharle et al., 2018; Fanelli, 2019; Geller et al., 2010; Resnik & Stewart, 2014). The good practices experienced in some institutions, of making education obligatory (at least for Ph.D. students) and embedding RI within competence models, offers inspiration across countries and disciplines. The importance also of complementary multilevel support within institutions in conjunction with educational measures puts the emphasis of responsibility for RI on institutions rather than individual researchers. Furthermore, the reported impetus for the development of RI education due to funders’ criteria suggests that European funders can influence the provision of RI in much the same way as the National Institutes of Health requirement of Responsible Conduct of Research (RCR) training for award recipients has influenced the development and provision of RCR education in the States (NIH, 2009).

### Infrastructure, Technology and Tools Supporting Daily Practice

The participants’ lack of preparations for the new GDPR legislation, demonstrates the importance of adequate infrastructure and tools for daily practice, as well as the complexity of implementing European regulations across different countries and research settings. Indeed, participants’ appreciation of infrastructure and tools integrated into daily practice reflects a more general need to reduce the complexity of performing research in the context of ever-increasing expectations of researchers. This need is recognized at a European level, with several European Commission funded initiatives aimed at making tools, standards, and protocols more readily available and accessible for researchers (Editorial, 2019; Labib et al., 2020). Technological or organisational tools, whilst helpful, may however prove to be inadequate if they are considered the main solution to complex RI problems, rather than part of a whole institutional approach.

### Strengths and Limitations

A study strength is its focus on the experiences of current RI support initiatives of a relatively large multi-disciplinary group of stakeholders at diverse levels of career development and the first of a kind comparison of experiences across three European countries. Comparing three countries also offers some insights and opportunities for mutual learning. That said, a limitation is that the themes identified undoubtedly have been influenced by the country selection. Comparing three countries with diverse levels of research and innovation activities may have resulted in more basic RI support needs being identified in Croatia and, to a lesser extent, in Spain, as well as giving a too positive impression of ‘good’ performance in the Netherlands. Furthermore, participants self-selected to participate, therefore the initial sample contained many RE + RI experts, although attempts were made to recruit younger researchers without a specific interest in RI in later rounds. Another consideration is that the consultation explored RI and RE issues, and preferences for online support. The study showed, however, that actually, for many participants, it was not possible to separate the concept of RI from RE. Furthermore, the relatively large number of participants (59) and the multiple rounds of focus groups, allowed for in-depth exploration and data of sufficient richness specifically on RI support in Dutch, Spanish and Croatian universities.

## Conclusions

This study highlights differences in experiences of RI support in three European countries in: the proactiveness of institutional RI governance and oversight, the extent to which RI oversight is a responsibility of RE structures, and the availability of support activities close to research practice. The study also reinforces the importance of a whole institutional approach to RI, embedded within local jurisdictions, rules, and practices. Such an approach puts the emphasis of responsibility for RI on institutions rather than individual researchers. If lacking, some stakeholders look for intervention by authorities, such as funders, outside of the university.

## Supplementary Information

Below is the link to the electronic supplementary material.

Supplementary file1 (DOCX 20 kb)

Supplementary file2 (DOCX 54 kb)

Supplementary file3 (DOCX 34 kb)

## Data Availability

Focus group transcripts are not available (in line with the promise of confidentiality in the participant information letter). However, the study protocol and information provided to participants is available here: https://osf.io/ezxpm/.
