# Supplementary material for: Stakeholders’ Experiences of Research Integrity Support in Universities: A Qualitative Study in Three European Countries
Source: Sci Eng Ethics. 2022 Aug 30;28(5):43. doi: 10.1007/s11948-022-00390-5 (PMC9427880; doi:10.1007/s11948-022-00390-5)
Supplement: Supplementary file 1 — Supplementary file1 (DOCX 20 kb) [file 11948_2022_390_MOESM1_ESM.docx]

# Topic guides

## Round 1

Introduction **10 min**

Introductory round

Presentation of the project and plan of consultation

**Topic 1. Understanding of RE+RI 20 min**

What do you understand by the terms RE +RI?

Possible probe – where is there overlap? How are they distinct

**Topic 2. RE+RI support available 30 min**

Tell me about the kinds of support for RI/good research practice and RE that exist in your work place?

*Possible probe - Can you tell me about the approach in:*

- *Guidelines*
- *Training*
- *Role models*
- *Other roles and structures*

*Possible probe - Do you think RE+RI is approached differently in different disciplines/sectors?*

**Topic 3. RE+RI unmet needs 30 min**

What kinds of support are currently insufficient?

*Possible probes:*

*Are the current support measures appropriate to your discipline/sector?*

*Can you find it?*

- *How should support be offered?*
- *How can we support you through the online platform?*

**Topic 4. Experiences 30 min**

Tell me about a time when you witnessed research that did not meet what you consider good research practice?

Why was that?

How could that situation have been avoided/the researcher supported?

## Round 2

Feedback **15 min**

- Feedback to participants about the themes identified from the first focus group
- How do these themes reflect or not reflect the discussion we had last week?
- Is there anything you would change/add?
- Presentation about the website and the preliminary collection categories

**Topic 1. We would like to understand your perspective better 15 min**

What would be reasons for you to visit the future website?

*Possible probe*

*If you have a question on how to do good research, which steps do you currently take to find an answer online?*

**Topic 2.We would like to understand your needs better 20 min**

*Please take a look at Image 1 - Home*
Looking at this homepage sketch, how would you start your search on the website?

*Please look at Image 2 - Article*

Imagine you just read an article on good research practices on the website, would you be interested in the questions and answers of the community related to the information on the website, and why?

**Topic 3. We would like to know how to become a trusted source 25 min**

What kind of moderation do you trust?

*Examples:*

- *Topic editors are assigned from the community to approve content*
- *Content is regulated by all community members*
- *A professional editorial team checks the content*
- *There is no regulation of content*

*Please look at Image 2 – Article*

What information is important for making content trustworthy for you? (number 3)

*Examples:*

- *The author’s job title*
- *The author’s organisation, company or institution*
- *The author’s research topic*
- *The author’s publications*
- *The author’s career*
- *The author’s contribution history to the website*
- *The number of upvotes of the content*
- *The number of views of the content*
- *Other: _______*

**Topic 4. We would like to know how you would interact with the website 30 min**

What would prevent you from contributing your knowledge on the website?

*Possible probing questions:*

- *Please look at Image 2 – Article. Would you take the effort to edit or add to existing articles if you could? (number 4) Or add new information if you could? (number 5)*
- *What would be reasons or incentives to do this?*
- *Would you like to be able to ask (number 7) and answer questions (number 8) anonymously?*
- *Would you like others to be able to request you to answer a question? (number 9)*
- *Would you prefer to share your questions and answers with the whole community or privately with a specific person? (number 10)*
- *Do you think upvoting questions or answers is a good idea, and why? (number 11)*

**Topic 5. We would like to know how you would like to login 15 min**

What information would you be prepared to share publicly on the website?

*Examples*

- *Real name*
- *Professional role*
- *Institute, organisation or company*
- *Profile picture*
- *Academic degrees or education*
- *Link to publications*
- *Link to Linkedin, PubMed, ResearchGate or Google Scholar profile*

*Possible probing questions:*

*For what activities would you be willing to create an account or log-in?
Examples*

- *Access to articles*
- *Access to questions*
- *Access to answers*
- *Upvoting*
- *Adding questions*
- *Adding answers*
- *Editing or adding to existing articles*
- *Adding new articles*
- *Adding or removing tags*

*How would you prefer to create an account for the website?*

- *Log-in with the credentials of an external service like ORCID*
- *Log-in with the credentials of a social platform like Facebook, Linkedin or Twitter*
- *Log-in by creating a new account with your email, real name and password*

## Round 3

Break out session 1

What did you find interesting or surprising about the results from the three countries?

What issues discussed are important in your discipline or work setting?

Do you think there are any other country or discipline differences that haven’t been captured?

Break out session 2

After hearing the declaration for the website, and a little bit about the content, functions and training, what are your first thoughts?

What do you expect/want from such a platform?
