# Supplementary material for: Stakeholders’ Experiences of Research Integrity Support in Universities: A Qualitative Study in Three European Countries
Source: Sci Eng Ethics. 2022 Aug 30;28(5):43. doi: 10.1007/s11948-022-00390-5 (PMC9427880; doi:10.1007/s11948-022-00390-5)
Supplement: Supplementary file 2 — Supplementary file2 (DOCX 54 kb) [file 11948_2022_390_MOESM2_ESM.docx]

| **Category code** | **Description** | **Example quote** | ***Subcategory code*** | **Description of subcode** | **NL** | **ES** | **HR** |
| --- | --- | --- | --- | --- | --- | --- | --- |
| ***1. Research Integrity (RI) governance and institutional implementation*** | RI governance and institutional implementation. Code category used if referring in general to norms and guidance. If type of guidance specified or named, subcodes are used. | **I:** Is there any other support that you would like for your faculty and your discipline that you don't currently have? **P43:** Well, I think it's a very broad field. There are many codes and regulation, very different types of problems.  **P43, Male, Researcher, Journal Editor, and RE or RI Committee Member, The Netherlands** | ***Codes, policies and guidelines*** | Codes, guidelines, and standards. **Including:** 1) descriptions of national, institutional or discipline specific codes, guidelines, standards and statements; 2) the need for such guidance; 3) perceived awareness of guidance. **Excluding:** 1) European and national laws. These are coded under 'legislation'; 2) Protocols and instructions for specific tasks in research practice. These are coded under 'Infrastructure, technology and tools supporting daily practice'>'other tools and tech'; 3) Institutional responsibility for RI guidance and efforts to translate guidance into practice, these are coded under institutional responsibility and implementation.  (code occurrence – 86) | **P4:** But, as an institute, and I know other academic hospitals have the same, we also have the whole institute research code on how to perform research in an ethical, open, honest, reliable – what are all the words? Honest, reliable, objective, impartially independent, open, fairness, responsible, and then all different topics covering that. And most universities, academic medical hospitals, have such a code. **I:** And the researchers doing the practical, day-to-day work? How well do they know the code? How are they made aware of it? **P4:** Can always be better. But I'm not sure if all academic medical hospitals function in this way, but when you get hired at the medical center, you get the general [Anonymized] – this is for [Anonymized] – code, which then can refer to all the different codes, like the research code. And it's on internet, it's on intra-net, yeah. **P3:** But it's hard to find if you Google it. **P1:** Yeah, that's the problem. **P3:** My PhD's can't find it – that's why we have this document with all the right links. Intra-net is chaotic. **P4:** That's true.  **P1, Female, Researcher, the Netherlands**  **P3, Female, Researcher, the Netherlands**  **P4, Female, Policy-maker, the Netherlands** | **P16:** In theory, at least in most centers, every time a new person arrives, they're given the code. **P18:** But I remember I received this and [hand gestures to throw it away]  **P16:** Of course, that's the problem. But I mean at least, when you arrive, you're given the code, you're told, you know, this is the website and that, but I agree, we need other ways of [multiple voices: yeah]. It's not only giving them the code, but say look, this is an essential part of your stay here.  **P18:** I think this is all good, and when I do things and I do them in similar ways, but I'm now very critical, because I know myself how these, what impact it has on me, and the policy I only read, then I need to edit it or if I want to make a new policy I need to refer to it, before if I didn't read the policy on I don't know, intellectual property or whatever, you know, I know it's there, and nobody reads, only if you are in a case, where you have to solve something specific.  **P16, Female, RE or RI Committee Member and Other, Spain**  **P18, Female, Research Manager, Spain** | [W]e have a code of conduct which was established in 2006, basically, so in Croatia it isn't a problem to have everything set up on the place, it's a problem with how this is working in reality. So that is the problem. When you have corruption, when you have politics which is going inside RI, inside the science system, and know that this is a problem in Croatia.  **P34, Male, Researcher and RE or RI Committee Member, Croatia** |
|  |  |  | ***Legislation*** | Legislation. **Including:**  1) Existing laws or legal frameworks; 2) desired laws or legal frameworks; 3) perceived awareness of legislation covering RI. **Excluding:** non-legally binding codes, guidelines, statements. These are coded under 'Codes, policies and guidelines'.  (code occurrence – 30) | **P13:** [i]t's the responsibility of the academic community to act as the conscience for the academic community. Now you were talking about legal embedding, and in that view, of which I'm also not convinced yet, but in that view of, it's the academic conscience, that is not necessary. There might be legal proceedings outside of that, that take this into account and say, OK, so the academic conscious can predict this, do we discipline him or do we disagree? So, I'm wondering if, how it works in different countries, and if this a relevant viewpoint at all, that I kind of feel, especially also given what's happening in Croatia, that, should we embed it in law? Maybe it doesn't have to be at all. Maybe it only has to be embedded in the academic community.  **P13, Male, Researcher, RE or RI Committee Member, The Netherlands** | So, I think there are flashes of integrity laws or integrity guidelines covering all of us [European level guidance]. The thing is that, you have the law but you don't know how to apply the law or how to implement that law in your way of working.  **P24, Male, Researcher, RE or RI committee member, and policy-maker, Spain** | **P33:** But again, data protection, including personal data protection, is law. So, this is not – you cannot *not* abide by law. You know, you can try and do research that does not abide by law, but then eventually there should be an institution that says 'hm, this does not abide by law, you cannot do this.' So yes, I think it's an incredible thing that finally we have started talking about data protection. And the Data Protection Agency has been here for 15 years, and it has done very little, in the last 15 years.  **P33, Male, Researcher, RE or RI Committee Member, Croatia** |
|  |  |  | ***Institutional responsibility and implementation*** | Institutional responsibility for, and implementation of, RI guidance. **Including:** the efforts made, lacking, and/or needed to ¨turn words into actions” to ensure guidance influences practice. **Excluding:** Individual researchers’ awareness and perceptions of guidance. This is coded under the specific type of guidance.  (code occurrence – 68) | **P13:** At our institution - it's the European Guideline on Data Protection - and it's making  quite a few difficult rules on basically how to treat privacy and personal data. At our  institution there's an awareness campaign. There's a group and they put things in your  physical mailbox, like they put, last time, a small box full of drop [Dutch liquorice] and they  called it 'drop box'. But the idea is more to keep pushing people, and this is what I was  thinking maybe in your institutions there is initiatives around these, trying to get people aware of the privacy aspects because it's now becoming an institutional role. **P11:** Just started a working group this week. [Laughter] **P13:** That's hilarious [considering the closeness of the legislation’s enactment.  **P11, Female, Researcher and Journal Editor or Assistant Editor, and RE or RI committee  member, the Netherlands**  **P13, Male, Researcher and RE or RI Committee Member, the Netherlands** | **P18:** You need the directors of the centers, or the manager or whatever, to actually be interested in this and say and “I'm going to, every year when I do the annual talk to my whole center, I'm going to touch on the issue of RI, and gender” [which was discussed as another cross-cutting issue], just to show that it is important. **P18, Female, Research Manager, Spain** | **P30:** So, in the vision there is a declaration that we would like to be integrated in European Research Area, and if you want to be integrated it has to comply with the principles. And then, okay, it's then again European standards, then we have this declaration, 'the University of [Anonymized] sees itself as a social leader which advocates and promotes the highest moral, ethical, social and economic principles and standards.' **P27:** So that's a strategy. Where are you reading from, it's the strategy? **P30:** It's the strategy, I said from the beginning. **P27:** Do you have it in action point? Do you it in action points? **P30:** No, we don't have action points for this one.  **P27, Male, Researcher, Croatia**  **P30, Female, Research manager and Policymaker, Croatia**  So, there is, when there is no relationship between words and deeds, like you say ‘action plans’ and everything, this is even worse than having nothing because you can show the people were doing wrong things, you can show 'look, we have very nice documents'.  **P34, Male, Researcher and RE or RI Committee Member, Croatia** |
| ***2. RI roles and structures*** | RI roles and structures. Subcodes only are used. |  | ***Committees*** | Committees. **Including:** discussion of RE+RI committees (coded together because they are the same committee in ES and HR); Participants' experiences with, and opinions about, RE+RI committees. **Excluding:** Other committees, such as scientific committees or advisory boards that provide advice. These are coded under 'Expert advice and contacts'.  (code occurrence – 87) | **P2:** We had a trial, and it was finished, so then of course, you let all the institutions know that you are finished, then you get information back what you should do now. So, the privacy officer, he said 'well, you should immediately destroy the connection between your persons and data. You should immediately destroy the connection.' And that surprised us. So, then we called our monitor from the Clinical Research Bureau, and they said, 'oh, we don't have an opinion, just do what the privacy officer says.' And then we thought, 'well, what would the medical ethical [RE] committee have to say?' And they said, 'no, you should save it for five years.' So then, yeah, well, what do you do?  **P2, Female, Researcher, the Netherlands**  Actually it's my personal opinion about how we're dealing with this RI is quite critical to how we're dealing with cases of research misconduct and one of the reasons is that you let these peers deal with these cases but they have no actual skills to do that, they're just professors who voluntarily, or sort of less voluntarily, applied to do it and who do it on top of their job without any training, without any guidance, without any support actually. **P10, Female, RE or RI Committee Member, and Policy Maker, The Netherlands**  **I:** And do you have a specific committee...? **P43:** On integrity, quality of research projects, on integrity, and since, I think two years, on ethics.  **P1:** Separate, yes? **P43:** No, we have just one committee. **P1:** One committee for both? **P43:** For both. Because we couldn't – we were unable to split it up. **I:** But that's specific to the law faculty?  **P43:** Specific to the law faculty. And then there's of course the committee on the university level.  **P1, Female, Researcher, the Netherlands**  **P43, Male, Researcher, Journal editor or assistant editor, RE or RI Committee Member, the Netherlands** | **P16:** So, it goes through a committee and they discuss it and they're looking for if its ok.  **P19:** I guess so. **P16:** Do they respond quickly? **P19:** because it gets done very quickly. Yes, they have two meetings per month, so it's, er, it's quite quick, and, yeah, the first protocol that we sent in the context of that project was to conduct interviews on the very, let's say on the methodological aspects of participatory research, so no sensitive data, it was not related to to patients or so, so it was very easy, but then for example a previous project that I was involved in on [an aspect of global health – anonymized] research, so the coordination is happening in Spain, but then you have the few working in it was [three low income countries - anonymized] and so yeah, it's validated, and you say, you state that you will be perform to work in a certain way, and then the resources aren't what they are... **P16:** And there's no training for the training for the people who are in the field at least in these aspects. **P19:** No, at least from mine…no. No, training on it. **I:** And no one to go to if you have a doubt after the ethics committee? **P19:** No. I guess you can call the ethics committee but it's not easy to, you can call the secretariat, you can ask to forward a question or, but I can't say because I haven't, I have never gone through that challenge. **P16, Female, RE or RI Committee Member and Other, Spain**  **P19, Female, Researcher and Research Manager or Administrator, Spain**  **I:** There is a follow up and within one year there is a specific control of the research committee to the research team in Spain, this is mandatory. **P21:** But professional doubt is difficult to ask to the committee. It is more easier to ask to the promotor, from the promotor of the pharmaceutical industry, or… But its, but I think it’s possible to ask but, yes.  **P21, Male, Researcher and RE or RI Committee Member, Spain** | **P28:** For instance, there was a case where some results were published in a bad journal and then sent to a good journal and it was claimed that the number of data was much much bigger, but the curves are completely the same. You cannot have the same average and the same statistical error; it was 10 times or 100 times bigger number of data. This was 100% proof that this was wrong. Nevertheless, other members of the committee did not want to prosecute or to make any strong decision, and the complaint came from the colleagues. **P28, Male, Researcher and RE or RI Committee**  [w]hat we [Croatian Committee on Ethics] are doing now, is an advisory board, so we are not sanctioning anybody. We are just providing opinions on some misconduct which are reported to us, not to go into details. So, we cannot sanction anybody, we are just providing opinions. **P34, Male, Researcher and RE or RI Committee Member, Croatia** |
|  |  |  | ***Expert advice and contacts (within universities)*** | Expert advice and contacts (within universities). **Including:** discussion of individuals - actual or desired - with a specific role to support RI within institutions (e.g. research integrity officers, research managers, librarians). **Excluding:** discussion of individuals with a specific role to support RI outside of universities, e.g. from national bodies, funding organisations, and independent consultancies.  (code occurrence – 35) | **P7:** Maybe there is a need of training, but also maybe a need of a person who can address to, to have these single questions. And now you have, I think, in University, of Confidential Counselor, but mostly you go there when you want to report something, or there might be an issue at the institutional level, but just information on.  **P7, Female, Working for a research funding organisation, the Netherlands**  Well, we have the course in, I think we went for three years now for all Ph.D. students and we advise them to do it in the second year. We have a plagiarism check with the thesis, of course we have the Research Integrity Officer and we have a Research Integrity Committee at central level and some departments have their own Research Integrity Officer. Some research schools also have them. We have... I think a couple of them, because we had a huge case in the media, Professor [Anonymized]'s case, so Ph.D. course came out of that. And we have, it's a couple of years ago, all departments have to write their own plan, how they want to be active with research integrity in their own departments, during meetings, where their weak spots are. So we did a couple of years ago but it's not that active at this moment any more.  **P15, Female Policymaker, the Netherlands** | **P16:** And then there's a good scientific practice working group, which is, this is the one we are part of, well there's two members of each center, and then we meet once every two months, or something like that, and then we discuss, so we talk about what each center is doing[...] And this committee, ah, it's not a committee, like a working group, is the only contact point that people in the centers have, like if the have a any problems… **P17:** they can go to these people **P16:** the person from their center, that is part of this group. And from there they direct to whatever, like, the center normally has their own ways of dealing with things.  **P16, Female, RE or RI Committee Member and Other, Spain**  **P17, Female, RE or RI Committee Member, Spain**  I mean I think it would be very good to have in each institution, one person that is the expert and that is dedicated to that. I mean we have this contact people, but they're just researchers that have been put there, and you know, they're now working as a contact person but I mean it would be, for example in the UK, like all universities have their research integrity offices, but here, there's no that kind of thing. Like there's no national research integrity office, etc. [agreeing noises]. So I think it would be good to have that.  **P16, Female, RE or RI Committee Member and Other Role, Spain** | **P34:** [y]ou can have one person on university, or whatever, or few persons, to whom you are approaching regarding research integrity issues. So maybe these persons should be fully independent, first, and educated in this research, should be an expert in this field. This is also a way how this can be solved, and I believe this can be even in the law, for example. You can put in the law, that university should have optimum person who will deal, and who will be independent. So that means also another way of – regarding in Germany or maybe some other European countries are – there are persons like this, in the Netherlands.  **P34, Male, Researcher and RE or RI Committee Member, Croatia** |
| ***3a. RI education*** | Code category used if referring generally to education or the experience of having received RI education that doesn't refer to the subcategories. For sections on specific subcode content, these subcodes are used. | **P48:** Well in my case, yeah, I started last year so I took these courses last year when I started. And for me it was very surprising because I learnt a lot of very new things as I was working and I realised that some things that I didn't expect happened and I could focus more on what we learned in these courses, no?  **P48, Female, Researcher, Spain** | ***Content*** | Content of the training. **Including:** topics that should be covered in all trainings or for specific disciplines. **Excluding:** how training should be delivered, these are coded under 'Delivery'.  (code occurrence – 17) | **P57:** [I] teach research integrity, and at first people think about these major cases, but they also feel like, well that's not happening in my department or in my discipline. But we also organised discussion groups and moral case deliberations and then we focus on the dilemmas that they experience themselves. And at first people say well, yeah, I don't really have a case, but then we give a few examples, and then everybody recognises it, so they all have a case. And then you really notice a shift in how they participate. And I think that is really important.  **P57, Female, Researcher and Other, the Netherlands** | **P16**: And again, as we're teaching this, I think most researchers also see also as a - it's a boring, you know, they can see the use of the statistics course. It's boring also, but they needs statistics. But I don't think they feel like they need research integrity. So in general I think we're happy, because the students tend to be very happy with the course, maybe because it's done through case studies and role plays, so it is interactive and they have fun. But the topic itself is, and that's students, if you try to get, you know, PI's, to go and talk, do anything about research integrity, I think it's even less likely that they're interested in it.  **P16, Female, RE or RI Committee Member and Other Role, Spain** | **P36:** Maybe as an illustration, we have done recently small research searching for instructions, education, and guidelines, on plagiarism, which is very hot topic in Croatia. And what is typical in Croatia, is that we find a lot of regulations, and lot of consequences, so 'if you will plagiarize, then you will, you know, something will fall from the sky and kill you.' But actually, nothing is happening, and there are no actually educational materials, they are not guidelines, anything, but just some kind of rules and threats, that's all what we find. And we compared it to international university, and you can see almost immediately how they are trying really to educate, not to blame someone.  **P36, Female, Researcher and RE or RI Committee Member, Croatia**  **P35:** We deal with issues once we have the huge cases and then we look at how we're going to prevent those in the future, how we're going to publish those in the future. But not having every student get all these moral dilemmas as a student thing. They're not in there. It's usually how you organise yourself around the university, but not how issues you may encounter when you first want to publish your paper with your mentor, who's going to be first author, or the second author, or, what if you have a disagreement with other researchers? Those are not included in these everyday preventative or introductory courses, and I think that's what we need to change.  **P35, Male, Researcher, Croatia** |
|  |  |  | ***Delivery*** | The delivery of training. **Including**: 1) delivery of training (e.g. stand-alone courses or embedded within other courses); mandatory or voluntary; who gives the training (e.g. trained trainers/volunteers); target audience (e.g. level and discipline); 2) the absence of training; and 3) initiatives to stimulate training at a national or EU level. **Excluding:** specific theoretical/conceptual approach and/or content of the course.  (code occurrence – 30) | At my university we made research integrity a learning line in our competence model, so every course has to think about what issues of research integrity do we want to address to our PhD students. **P10, Female, RE or RI Committee Member and Policy-maker, the Netherlands**  **P14:** Also, I'm not sure if we will get it compulsory for more senior staff but I really would like, and maybe we have to incorporate in to something else, what's compulsory for them so that's not thinking 'oh, I have to do something'. **I:** Do it sneaky. **P14:** Yeah, do it the sneaky way **P14, Female, Researcher, the Netherlands**  Well, sometimes, but usually only at the level of the university. Then we have now and then meetings, and some lectures, especially to teach PhD students. I think our big problem – I think that's everywhere – the senior staff, how can you reach the senior staff members? The youth isn't the big problem, I think. **P43, Male, Researcher, Journal Editor or Associate Editor, and RE or RI Committee Member, The Netherlands** | And I think, I mean this course has been going on since 8 years, or maybe more […] like last year already I was asked to give a course on research integrity at a different institution, and this year I got six different institutions in Barcelona asking for, so they have never done anything, and now they're, they see the need, but they're being forced by you know, the European Community Commission, or funding agency or whatever. So, I think it's definitely changing, but I'm not sure if it's because they see the need or because they're being forced a little bit.  **P16, Female, RE or RI Committee Member, Spain**  So, you need to train your researchers, but you also need to train the manager because, well, you interact with the researchers and you need to talk the same language.  **P19, Female, Researcher and Research Manager, Spain** | What I see as a help is to create, for example, educational materials which can be easily implemented in the courses we already have, which will be actually adapted for our academic community […] Of course, guidelines and instructions, but not primarily just that, because when I was actually introducing to Croatian editors some European standards 15 years ago, but you can count on the fingers of one hand how many editors read that guidelines. So, we really need a lot of materials, a lot of support, for example presenters, people coming to the universities, and giving lectures for students, giving lectures for university management, and that's important.  **P36, Female, Researcher and RE or RI Committee Member, Croatia**  **I:** Do you have to have training then? Do you have to follow these trainings or are they voluntary? […] **P32:** They are completely voluntary.  **P30:** Unfortunately.  I: How about for PhD students?  **P30:** Still voluntary.  **P32:** Some... like, one grad school programme has the research integrity part included. Other two PhD programmes, I think they do not.  **P30, Female, Research Manager and Policy Maker, Croatia  P32, Male, Researcher, Research Manager, and Journal Editor or Assistant Editor, Croatia**  I honestly think that we can make a difference with schools and seminars to which senior staff should also be obligated. I honestly think so because they didn't get this formal education. **P29, Researcher and Journal Editor or Assistant Editor, Croatia** |
|  |  |  | ***Combined with other support*** | The other types of support that are important to implement in combination with training. **Including:** specific types of support needed (e.g. protocols, guidelines, codes of conduct, good role models) or general mention of supportive infrastructure that needs to be in place without mention of specific initiatives. **Excluding:** Discussion of other types of support if not described ain combination with training.  (code occurrence – 18) | **P7:** Maybe there is a need of training, but also maybe a need of a person who can address to, to have these single questions.  **P7, Female, Funder, the Netherlands**  **P14:** As an organisation or institution you want to make research as easy as possible to do in the right way and you need to facilitate in infrastructure and in education and in mentorship and so on.  **P14, Female, Researcher, the Netherlands** | I just feel that we receive a training in our first year and, I'm in my fourth year, honestly, I don't remember almost anything of this course and I think, just thinking from an institutional point of view, I feel there's a gap of protocols, for example. There should be a protocol how to manage data, there should be a protocol, something very systematic that should follow, yes, some good practice guidelines. **P50, Female, Researcher, Spain** | We tend to put the blame on the individual and take it a long way from the responsibilities of universities. And I think when you approach them as, when you teach research integrity, ideally you want the right resources to prevent this at the university/institute level, that could prevent data manipulation, that could have to do with data storage.  **P35, Male, Researcher, Croatia** |
|  |  |  | ***Cases, casuistry, and scenarios*** | Cases, casuistry and scenarios. **Including:** participants preferences for the types of cases, casuistry and scenarios important for teaching and for the platform. **Excluding:** discussion of actual or suspected cases of poor research behaviour.  (code occurrence – 34) | **P15:** And then I'm looking for information that can help me in my meetings with researchers who are struggling with an issue themselves or who want to make an official allegation of accusing somebody else of misconduct or whatsoever, just to be, well – that I have enough arguments or experience to do the right thing and to help them. **P15, Female, Policymaker and Other, the Netherlands** | **I:** That's definitely an issue for PhD students. Why would you go to such a website? **P17:** Maybe case studies and yeah, courses and materials that we can use for the courses. **I:** What specific materials would you like on those? **P17:** It's the study activities that we can incorporate in our seminars, and things that we can incorporate. **P16:** I think case studies and real examples. **I:** What are real examples about? **P16:** Real examples of things that went wrong, good point, not about 'proper misconduct' as in the big scandals we all know, but real examples of people saying look what happened to me. I did this and I didn't realise and I didn't name the file properly and then this happened and I lost all the data. Real examples of real people, things that could happen to anyone, if you are not careful enough. I think that would be really good and nothing matches that anywhere.  **P16, Female, RE or RI Committee Member and Other, Spain**  **P17, Female, RE or RI Committee Member, Spain** | **P33:** On cases, both theoretical cases and true cases, and how to deal with those ethically. Sort of like, algorithms, or code paths, or when they, someone, the reader, has come up with something to complain about an article that probably has some plagiarism to the editor, and how to proceed from there. Or, that a reviewer has complained to the editor, or a reader has complained [.....] algorithms of ways of how to deal with these things ethically. And everyone involved in this is what we can expect as an outcome, but I think that the researchers themselves who are interested in these topics will benefit.  **P33, Male, Researcher and RE or RI Committee Member, Croatia** |
|  |  |  | ***Reflexive practice*** | Reflexive practice. **Including:** sections where participants describe or identify a need for reflexive practice are coded under ¨Reflexive practice¨. Not as part of a course content but rather as a desirable habit of researchers. Also coded here the ability of the academic community to regulate itself and the measures needed for that. **Excluding:** specific course content to encourage reflexive practice, this would be coded under 'content'.  (code occurrence – 24) | **P10:** so I think if you were to evaluate research integrity, it shouldn't be just about how will you avoid people, or how you respond when your data, or parts of research data is being fabricated or whatever, it should also be, how will I make extra efforts to increase the quality of your research, because I think that is the core issue of research integrity also. **P33:** Just a quick comment. Whenever we say extra, then that really trickles down into the researcher, as, oh, "extra" work.  **P10:** Yeah, I know. I think if you were to put it, and I don't have the full answer on that as well – if you were to put it in an efficient way, it shouldn't be any extra work, it should be part of that critical, reflexive thing, and it should be like a second nature for researchers. But it's difficult to avoid making it a formalised form they need to fill in and administrative tasks they have to do. Yeah, that's the challenge  **P10, Female, RE or RI Committee Member and Policy-maker, the Netherlands**  **P33, Male, Researcher and RE or RI Committee Member, Croatia**  What I like about what you said before is, I kind of have the feel from talking to colleagues that they see research integrity as Ray [Co-author-RdV] put it, like, am I behaving badly? And you know, everyone says of themselves 'no', and that shuts the door on that, we're done. So you made a remark that made me think, yeah, if we can get people to consider integrity not as a yes or no question, but more as a broad spectrum, where also, it's not just the penalty - you misbehave or not, but for example, if you were saying, like, did you, in retrospect, looking back at your research, maybe you should have done it differently. It doesn't mean you lose. It doesn't mean now you're going to kiss your job goodbye or you know, disciplinary action. It just means you look back at it and say, that wasn't the way it should be. And I think that would help with selling it to researchers who are well-intended and incredibly pressed for their time already, and feel like ethics and integrity is, you know, ‘either I get smacked with a big hammer or not’.  **P13, Male, Researcher, RE or RI Committee Member, the Netherlands** | [w]e want that people take it as real, an issue, that they want to solve for their research, because it makes their research better, it makes them more happy, because they can trust on what they do, that it's okay and that it's good for society and everybody, and so in this I don't  see how this can be done. At the moment, to be honest, I don't really see how to change it at the moment, I don't feel it's in my hands, that we can change this culture at the moment with these issues, and it would be fantastic to have a tool or measure to change it. Uhm, yeah. I'm a bit unhappy with it at the moment. And there's not, I don't know if it is only related to training, maybe it can be solved, but I'm not sure. **P18, Female, Research Manager, Spain**  **P49:** For me it sounds good but it sounds bit like we are just doing this because we have pressure from the outside but we should also, from the inside, as good scientists, be like, now I want to do trustworthy replicable science. Data that can be replicated by others.  **P49, Female, Researcher, Spain** | See entry under NL for conversation between a Dutch and a Croatian participant. |
| ***3b. Supervisors and peers*** | Seniors and peers as sources of support. Subcodes only are used. |  | ***Seniors*** | Discussion of the influence of senior staff behaviour for research practice. **Including:** 1) seniors and mentors as a source of RE+RI advice; 2) senior staff as instigators of RI initiatives; 3) the influence of (good and bad) role models; 4) perceived deficits in knowledge and poor practices of senior researchers. **Excluding:** Non-research related behaviours.  (code occurrence – 52) | In these sessions, senior, but also the juniors, are stimulated to share like, for instance, if you published an article, and then in hindsight you realized 'oh my God, I did one of the analyses in the wrong way, what should I do?' So, we really want to stimulate that the juniors are not keeping it to themselves, but share them, and then we can cope with it all together. **P2, Female, Researcher, the Netherlands**  **P9**: Support is good coaching of the PhDs and even the students, let's say the undergraduate students, because they have their scientific activities. We like that, and then if you don't put it on the agenda then, it's very difficult to be credible later on. **P11:** Do you have specifically things or meetings or whatsoever to guarantee good mentorship? Because I think, well in our organisation it is an issue, to get really good mentorship for all. **P9**: Well, it depends whether it's my own department or the whole faculty, but in our  department we try to have a very intensive dialogue between seniors and also about their  coaching issues.  **P9, Male, Researcher and Journal Editor or Assistant Editor, the Netherlands**  **P11, Female, Researcher and Journal Editor or Assistant Editor, and RE or RI committee  member, the Netherlands** | **P52:** Mainly my own support and sometimes my supervisor but as one [missed] says, it's very interesting to see what you are doing. Everything you learnt from your supervisor. So, everything I'm doing is just from his point of view and now I can realise that there is some stuff that is not properly done but I'm starting to realise after 7 years. So, its a little late, for my PhD it's too late.  **P52, Female, Researcher, Spain** | **P30:** In all honesty, this is the lowest grade of evidence I know, and we always go to our senior colleagues who we know that have more experience and are expert at certain field and then we go to them. And then they can refer us to certain literature, web-pages or laws and so on. But usually, it's just by talking to our colleagues.  **P30, Female, Research Manager and Policy Maker, Croatia** |
|  |  |  | ***Peer support and behaviour*** | Peer support and behaviour. **Including:** 1) the influence of peers' behaviour; 2) peer RI support initiatives (instigated from the top down or be bottom up). **Excluding:** Non-research related behaviours.  (code occurrence – 15) | [n]ot formal things but really the informal discussion with other researchers, like how do other  people deal with, because I think we're all dealing with the same type of problems. **P14, Female, Researcher, the Netherlands** | **P52:** we are lucky because we have a very multinational team so we have people from Portugal and France and sometimes we discuss this stuff, but also it seems like everybody's lost. I don't, sometimes I start to feel like I don't trust anybody. I'm starting to think like this. After I see how you research, there are less people that I can trust, there are fewer people that I can trust, that I can trust my research or I can trust ok, you can collect the sample. I'm starting to feel a bit more like, I'm a bit lost, I don't trust people unless I can see that you are trustworthy for myself.  **P52, Female, Researcher, Spain** |  |
| **4. Infrastructure, technology and tools supporting daily practice** | Subcodes only are used. |  | ***Data management*** | Data management. **Including:** 1) Agencies, laws, codes, and guidelines related to data management; 2) Data collection (appropriateness, quantity and use); 3) Record keeping, including documentation of analysis decisions; 4) Completeness of data, including metadata; 5) Storage of data, including privacy/data protection issues; 6) Access to data (open or not); 7) Big data.  (code occurrence – 39) | [w]e have a huge department, it's called [name anonymised – the dept. involves engineering], there is some research done on automotives, which has a lot of different problems than the medical, the health department. They are more on data management, because they are gathering a lot of data with modern cars, they get a lot of data. So, their problems are quite different from the health department. […] No, because I walked in a few months ago, and I asked them what do you do about data management? 'Well, we just gather the data, and we play with it, we do a lot of crazy things with them.'  **P43, Male, Researcher, Journal Editor or Associate Editor, and RE or RI Committee Member, The Netherlands** | Actually, I think that's it, and for example, the point of data management is a very, very  important one and it's something that should be very structural because at the end, it's not just  how I use the data, it's also all the team that is going to be with the data, starting with the IT  people, statisticians and whatever, I think there's a very big gap of training to everyone.  **P50, Female, Researcher, Spain** | **P38:** I think the problem is different in academia and private research sector, because we have everything covered with policies, everything is controlled. Laboratory books, you can check every number and very fast you can find out if somebody makes a fault or something that is not ... **I:** And this is in the ... **P38:** In the – yes this is in the industry and in [company anonymized] and everything, because this is very important for patents. And of other things, you don't have so much freedom to choose which kind of research you will provide, so this gives you frame, and then you can put something in this in academia, is my opinion. So, it gives a lot of freedom of it, nobody checks your approach or if something is right. Can somebody check what you did with some experiments, how you provide data, is this something that is acceptable or not? How this works. I worked also in academia and then that was good research practice, that is similar to this policy in the private sector, but I don't know if this is still so...  **P38, Female, Researcher and Research Manager, Croatia** |
|  |  |  | ***Other tools and tech*** | Other tools and tech to support daily research practice. **Including:** descriptions of technology (e.g. software to check for plagiarism, check analyses, check duplicate articles), platforms (e.g. Github, PubPeer), protocols, SOPs to support good research practice. **Excluding:** Broader RI guidelines and codes. These are coded under 'Codes, guidelines, and standards'.  (code occurrence – 8) | **P13:** I'd like to react to the idea of having an agreement between supervisor and PhD student. As I said before, background in security, yeah, no. So, the institution can set guidelines on the agreement, but an agreement between someone who owns your job funding and can fire you, yeah, that's like a wolf and sheep voting what's for dinner. So, if there should be something done, there should be something at the institutional level. If you start to think about it, it has to be some generic terms, because cases will differ, so it will be something like, if you know the supervisor is involved in 10% of the work, or is responsible for 10%. And this will– I can immediately see that it might solve some things, it immediately leads to discussions again, like, oh but you didn't do 10%. So, I'm not convinced this is the best way.  **P13, Male, Researcher, RE or RI Committee Member, The Netherlands** | **P50:** But this eighty per cent [of papers which are not reproducible] includes very old papers? Because I think this is also changing all the time, no? This estimation is also taking papers from the 60s? Because I'm sure if you do these with papers from the last five years the percentages are going to be different, because we are also developing new tools and new ways of working and I think that this is a kind of evolving process. For example, that we use data, and these days its growing a lot R and using GitHub which is a kind of open space where you can share your codes, so I don't know.  **P50, Female, Researcher, Spain**  **P16:** No, I'm just thinking the type of cases that you can find yourself as an editor will be mostly, I mean my time as an editor was short and I did not have to face any of these issues fortunately, but I guess it's authorship problems and if someone contacts you and says look, I have misgivings about this article, or obviously now, PubPeer, so you get an email from PubPeer, saying an article published in your journal has this duplicated image blablabla, and I don't know if journals have, I guess each journal has their own protocol on what to do in those cases, whether they contact the institution or not, as long as I know the journals don't do any investigations or anything, they contact in any case, the institutions where the researchers are and ask, you know, can you sort this out? But I don't know if that's a common way of all the journals to do it. Or but yeah I think-  **P16, Female, RE or RI Committee Member and Other, Spain** | I know many of institutions, also this one, but [Institution anonymized], did have this software program for plagiarism. It is small step forward, but it is. It does not prove if it's really plagiarism or not, but it's some kind of checking, so it's telling you you should be aware that something will be checked, the first time.  **P37, Male, Researcher, Research Manager, and Journal Editor or Associate Editor, Croatia** |
| None | Codes that are not subcodes of the four categories, but which cut across categories. |  | ***Discipline/domain differences*** | Discipline/domain differences. **Including:** 1) issues specific to particular research disciplines; 2) comparisons between research disciplines; 3) comparisons between research domains (e.g. industry compared with academia, undergrad vs. post grad research, research in universities of applied science vs. research universities).  (code occurrence – 65) | **P43:** we are building a system but not on our own, but on the level of the university, a system of gathering and storing data, and data protection. Because we are legal scientists, but that's not only a black letter science, it's also about interviewing people, etc. And then, it's social science, so we have to deal with the same problems as our social science faculty. But we are not very aware of that, so we have to learn very much in this field. That's one of our problems.  **P43, Male, Researcher, Journal editor or assistant editor, RE or RI Committee Member, the Netherlands** | **P50:** We were very aware about going through an ethics committee, about writing protocols, about data management. Comparing with other stakeholders who were maybe from countries that we, from a prejudice let's say, we could think they were more strict, from Northern Europe. And they were less because they were from another field […] and they were not so used to working with participants on things like that.  **P50, Female, Researcher, Spain**  **P51:** I think that there is heterogeneity, in research, research in my context for instance, I'm doing clinical research in the hospital and in the end we have to go to the committee of ethics of the institution and you have this support, right? Of course, there could also be a misconduct of the research. You always have a phone to ask how to do the things, how to match the data, you know, so in this case I think that I have noticed that it is true that, most of the people in my context start doing research after their residence when they, to become a doctor, they almost don't know almost nothing about ethics integrity of research so, this is the situation at the hospital. **I:** And because they're clinical doctors they seem to be- **P51:** There is not any course of any other degree when you are becoming a doctor approaching this aspect of research, this aspect of ethics and integrity.  **I:** What would you like to see? **P51:** I would like to see that the institution be more supportive in this kind of, at least in people that is doing their residence, that in the end, in a hospital, in a university hospital, about eighty per cent of people that are doing their residence will start after the residence doing research or clinical research. So, some kind of specific support for teaching these people at the hospitals would be necessary. **P51, Female, Researcher, Journal editor or assistant editor, Spain** | **P33:** When we discuss these things, these go beyond just whether we are dealing with animals, humans, cells, and personal data, and sensitive data in an ethical way, these also include the way that we publish, if we don't publish. So I just want to reframe the question between academia and industry. While the industry has to follow strictly their policies, those policies do not necessarily include publishing their data. And just think how recently, how much ... how much effort the pharma industry created to oppose the ideas of having to actually register every single file.  **P38:** This is clinical part, research part is something else. That is also problem. But we can check every data, but this is something that ...  **P33:** I know you can check that in the academia journals, but this goes beyond just whether you can check the data.  **P38:** Or if it is necessary.  **P37:** But also I have the [.....] in industry, or in the private sector, they are more focused on protecting of intellectual property, and data. So how this apply, on this purpose, to have research integrity and research ethics in patent writing, or patent protection, that is something what is not available for a broad publicity.  **P33:** Obviously, so one side we have the, for example, the stakeholders and the stockholders that you are responsible to, because you have to prove that you are working towards a net plus, so you want your company to strive, while on the other hand, there is a lot of other things that maybe negatively influence the balance. So there is - it is a bit more complicated than just uh 'this is something that we need to patent and we will not show anyone our data.'  **P33, Male, Researcher and RE or RI Committee Member, Croatia**  **P37, Male, Researcher, Research manager, Journal editor or assistant editor**  **P38, Female, Researcher and Research Manager, Croatia** |
|  |  |  | ***Country context*** | Country specific factors. **Including**: 1) history; 2) culture; 3) national development of RI oversight. **Excluding:** National level guidance – this is coded under 'Codes, guidelines, and standards', or ‘Legislation’.  (code occurrence – 60) | **P41**: This is a very uniform country. **P2**: We all agree, every time... **P41**: We talked about it before... **P2, Female, Researcher, the Netherlands P41, Male, Researcher, Research Manager, Journal Editor or Assistant Editor, and RE or  RI Committee Member, the Netherlands** | **P54:** These kinds of tools, for me, because we use these kinds of tools in the practice of medicine, it's very important because you know that humans don't learn without, if the human don't make mistake, It's the only way to learn and to go forward. But Spanish never say ‘I have made a mistake’, its impossible. **I:** I think the idea of sharing the stakes is a really good learning opportunity.  **P54:** We feel very strongly that we don't say nothing, it's like our politicians, never, never, never. **I:** That's in every country. **P54:** Spain is different.  **P54, Female, RE or RI Committee Member, Spain** | So, what happened in Croatia, we have –with our country, since 1991, then we've had  socialism, then we had war, we've had everything, you know. And you are putting everything together, trying to put science inside of everything, and then putting research integrity or ethics or morality... During the war, for example, after the war... So we have had consequences of that. That is of course, my personal opinion, which are reflecting. And then before the war we were – like the whole Eastern Europe I believe, not just Croatia – we were  under socialism and there were other rules of morality, let's say, or of ethics, whatever,  which were also correlated to the political system of that time. So that's always correlated, also in our time, but at the time it was at a higher level, let's say. **P34, Male, Researcher and RE or RI Committee Member, Croatia** |
|  |  |  | ***Audits*** | RI audits and checks. **Including:** audits and checks on the conduct of research and research outcomes (data/code) by teams, institutions, funders, or software.  (code occurrence – 21) | **P13:** What I think will happen is something you see in mathematics already for a long time, there will be more of a push for openness and people will make meaningless things available, and they're meaningless because they aren’t checked, they aren't validated, they're not confronted that they actually even work, let alone work in the way that you described in the paper. And this is what I mean with meaningless, so I can make things open source as it's called, and then can put it on my website and I can say 'look, everyone can download it'. If no-one does, and no-one will because seriously, how... none of you have read my papers, right? Of course not. So, no-one is doing this. And you have the same with mathematics because for years they are talking there about, they want to go to computer verified proofs. Proofs in mathematics are hard and computers can automatically check all the nitty-gritty details and they want to get to this point, and they've been talking about that since the Eighties. And it's not the Eighties anymore and they're still talking about it but they're not doing it. And so, this is... so I do think there will be more available, I think this is basically fake. As in, this is hardly checked. **P14:** But it is at least possible to check. And that's the first step. **P15:** I think that nobody does it shouldn't be the reason for not making it transparent. **P13:** No, no, no, I did not want to say that. I completely agree, but I do think, you need something more to actually move beyond. So, I think the first step is everything should be available, but the second step should be we should also check. I mean, this is sort of the premise of science, right? We check things by other people and then we say it's okay, then it's published. That’s kind of the premise, and we're not doing that with data, with programmes, with things. We say they're available, we've read the paper.  **P13, Male, Researcher, RE or RI Committee Member, The Netherlands**  **P14, Female, Researcher, the Netherlands**  **P15, Female, Policymaker and Other, The Netherlands** | **P54:** Assessing what was happening after our approval, we have learnt that the investigators, the researchers, forgot all the things that they are going to do, all the talks that they are going to do, they have approval and they forgot all. Most of the research investigators, after two years they have to show us the works.  **I:** The outcomes?  **P54:** The outcomes. Because it's one of the most important things for us that they have planned it well, but they are [also] doing well and they are publishing well.  **I:** So, you actually follow them through the whole process? **P54:** We have begun two months ago only. **I:** Okay, and what is your experience of that? **P54:** Our experience is that like a wall, big high wall, they are, they have to learn because they have to explain that this public money has been well invested. **P54, Female, RE or RI Committee Member, Spain** | [b]ut for countries like ours where the institutions themselves do not necessarily follow their own guidelines or stick to the law, to the letter, I think it's important that we have also have outside audit. Discussing the Agency for the Protection of Data, and for reasons unknown the only thing that – only personal data is what they should be concerned with. They were instituted by Croatia having to listen to European law, so if eventually one day, European law says 'every country should have this and that', then you have to follow through, and eventually someone will check by an audit of some kind, of random audit, and we had better fulfil all these ... **P33, Male, Researcher and RE or RI Committee Member, Croatia**  Yes, so I think these things need to become common knowledge, and people need to be aware that there will be someone checking what they are doing. Hopefully as someone from the Croatian Science Foundation, or the funding body, the basically, the only funding body that we have. Ideally the funding body itself should have someone checking what is being done with their money, and not rely on someone else. […] So, with the creation of the National Science Fund, ideally, the oversight for some of these things, including the research  misconduct, should lie with the funder, not necessarily – the funder should not necessarily  rely on the corrupt policies of institutions who usually only have an ad hoc committee who  doesn't really know what they're doing. They just want to get rid of any implications that someone there might have done something wrong. **P33, Male, Researcher and RE or RI Committee Member, Croatia** |
