# Supplementary material for: Stakeholders’ Experiences of Research Integrity Support in Universities: A Qualitative Study in Three European Countries
Source: Sci Eng Ethics. 2022 Aug 30;28(5):43. doi: 10.1007/s11948-022-00390-5 (PMC9427880; doi:10.1007/s11948-022-00390-5)
Supplement: Supplementary file 3 — Supplementary file3 (DOCX 34 kb) [file 11948_2022_390_MOESM3_ESM.docx]

|  | Focus group round | | | | | |  |  |
| --- | --- | --- | --- | --- | --- | --- | --- | --- |
| Participants | **Round 1** | | **Round 2** | | **Round 3** | | **Across rounds** ^a^ | |
|  | **No** | **%** | **No** | **%** | **No** | **%** | **No** | **%** |
|  | **40** |  | **38** |  | **23** |  | **59** |  |
| **Country** |  |  |  |  |  |  |  |  |
| The Netherlands (NL) | 15 | 38 | 13 | 35 | 9 | 40 | **25** | **43** |
| Spain (ES) | 10 | 25 | 12 | 32 | 8 | 35 | **17** | **29** |
| Croatia (HR) | 15 | 38 | 13 | 35 | 6 | 27 | **17** | **29** |
| **Gender** |  |  |  |  |  |  |  |  |
| NL Female | 9 | 60 | 7 | 54 | 4 | 45 | 12 | 48 |
| NL Male | 6 | 40 | 6 | 47 | 5 | 56 | 13 | 52 |
| ES Female | 6 | 60 | 10 | 84 | 5 | 63 | 13 | 77 |
| ES Male | 4 | 40 | 2 | 17 | 3 | 38 | 4 | 24 |
| HR Female | 7 | 47 | 7 | 54 | 3 | 50 | 13 | 60 |
| HR Male | 8 | 54 | 6 | 47 | 3 | 50 | 9 | 41 |
| All Female | 22 | 55 | 24 | 64 | 12 | 53 | 26 | 45 |
| All Male | 18 | 45 | 14 | 37 | 11 | 48 | 33 | 56 |
| **Age** |  |  |  |  |  |  |  |  |
| NL 20-29 | 1 | 9 | 2 | 25 | 0 | 0 | 2 | 11 |
| NL 30-39 | 7 | 64 | 3 | 38 | 4 | 67 | 9 | 50 |
| NL 40-49 | 2 | 18 | 1 | 13 | 1 | 17 | 3 | 17 |
| NL 50-59 | 1 | 9 | 2 | 25 | 1 | 17 | 2 | 11 |
| NL 60-69 | 0 | 0 | 0 | 0 | 0 | 0 | 2 | 11 |
| ES 20-29 | 0 | 0 | 2 | 17 | 2 | 25 | 2 | 12 |
| ES 30-39 | 3 | 30 | 4 | 33 | 2 | 25 | 6 | 35 |
| ES 40-49 | 6 | 60 | 4 | 33 | 3 | 38 | 7 | 41 |
| ES 50-59 | 1 | 10 | 2 | 17 | 1 | 13 | 2 | 12 |
| ES 60-69 | 0 | 0 | 0 | 0 | 0 | 0 | 0 | 0 |
| HR 20-29 | 2 | 18 | 2 | 22 | 1 | 20 | 2 | 18 |
| HR 30-39 | 3 | 27 | 3 | 33 | 2 | 40 | 3 | 27 |
| HR 40-49 | 4 | 36 | 3 | 33 | 2 | 40 | 4 | 36 |
| HR 50-59 | 1 | 9 | 0 | 0 | 0 | 0 | 1 | 9 |
| HR 60-69 | 1 | 9 | 1 | 11 | 0 | 0 | 1 | 9 |
| All 20-29 | 3 | 9 | 6 | 21 | 3 | 16 | 6 | 13 |
| All 30-39 | 13 | 41 | 10 | 34 | 8 | 42 | 18 | 39 |
| All 40-49 | 12 | 38 | 8 | 28 | 6 | 32 | 14 | 30 |
| All 50-59 | 3 | 9 | 4 | 14 | 2 | 11 | 5 | 11 |
| All 60-69 | 1 | 3 | 1 | 3 | 0 | 0 | 3 | 7 |
| **Role** |  |  |  |  |  |  |  |  |
| *Researcher* |  | % |  | % |  | % |  | % |
| NL | 8 | 54 | 9 | 70 | 6 | 67 | 16 | 64 |
| ES | 6 | 60 | 8 | 67 | 7 | 88 | 12 | 71 |
| HR | 11 | 74 | 9 | 70 | 6 | 100 | 12 | 71 |
| All | 25 | 63 | 26 | 69 | 19 | 83 | 40 | 68 |
| *Member of a research ethics or research integrity committee* |  | % |  | % |  | % |  | % |
| NL | 6 | 40 | 2 | 16 | 3 | 34 | 8 | 32 |
| ES | 5 | 50 | 3 | 25 | 1 | 13 | 6 | 36 |
| HR | 3 | 20 | 3 | 24 | 3 | 50 | 4 | 24 |
| All | 14 | 35 | 8 | 22 | 7 | 31 | 18 | 31 |
| *Policy maker* |  |  |  |  |  |  |  |  |
| NL | 4 | 27 | 1 | 8 | 1 | 12 | 4 | 16 |
| ES | 1 | 10 | 0 | 0 | 1 | 13 | 1 | 6 |
| HR | 1 | 7 | 1 | 8 | 0 | 0 | 1 | 6 |
| All | 6 | 15 | 2 | 6 | 2 | 9 | 6 | 11 |
| *Research manager or administrator* |  |  |  |  |  |  |  |  |
| NL | 0 | 0 | 2 | 16 | 0 | 0 | 2 | 8 |
| ES | 2 | 20 | 0 | 0 | 1 | 13 | 2 | 12 |
| HR | 5 | 34 | 3 | 24 | 1 | 17 | 5 | 30 |
| All | 7 | 18 | 5 | 14 | 2 | 9 | 9 | 16 |
| *Journal editor or assistant editor* |  |  |  |  |  |  |  |  |
| NL | 3 | 20 | 2 | 16 | 0 | 0 | 5 | 20 |
| ES | 0 | 0 | 1 | 9 | 0 | 0 | 1 | 6 |
| HR | 4 | 27 | 4 | 31 | 3 | 50 | 5 | 30 |
| All | 7 | 18 | 7 | 19 | 3 | 14 | 11 | 19 |
| *Working for a research funding organisation* |  |  |  |  |  |  |  |  |
| NL | 2 | 14 | 2 | 16 | 1 | 12 | 2 | 8 |
| ES | 0 | 0 | 0 | 0 | 0 | 0 | 0 | 0 |
| HR | 1 | 7 | 2 | 16 | 0 | 0 | 2 | 12 |
| All | 3 | 8 | 4 | 11 | 1 | 5 | 4 | 7 |
| *Research policy, training, or compliance officer in industry* |  |  |  |  |  |  |  |  |
| NL | 0 | 0 | 0 | 0 | 1 | 12 | 1 | 4 |
| ES | 0 | 0 | 0 | 0 | 0 | 0 | 0 | 0 |
| HR | 1 | 7 | 0 | 0 | 0 | 0 | 1 | 6 |
| All | 1 | 3 | 0 | 0 | 1 | 5 | 2 | 4 |
| *Other* |  |  |  |  |  |  |  |  |
| NL | 1 | 7 | 1 | 8 | 1 | 12 | 2 | 8 |
| ES | 2 | 20 | 2 | 17 | 1 | 13 | 2 | 12 |
| HR | 0 | 0 | 0 | 0 | 0 | 0 | 0 | 0 |
| All | 3 | 8 | 3 | 8 | 2 | 9 | 4 | 7 |
| **Highest departmental position (if applicable)** |  |  |  |  |  |  |  |  |
| ***Netherlands*** |  |  |  |  |  |  |  |  |
| PhD student | 1 | 7 | 3 | 24 | 1 | 12 | 4 | 16 |
| Mid-career (Post doc, senior researcher, assit. or assoc. prof) | 5 | 34 | 3 | 24 | 4 | 45 | 7 | 28 |
| Late-career (Prof and Heads of dept) | 2 | 14 | 1 | 8 | 1 | 12 | 3 | 12 |
| Missing | 0 | 0 | 2 | 16 | 0 | 0 | 2 | 8 |
| ***Spain*** |  |  |  |  |  |  |  |  |
| PhD student | 1 | 10 | 3 | 25 | 4 | 50 | 4 | 24 |
| Mid-career (Post doc, senior researcher, assit. or assoc. prof) | 4 | 40 | 5 | 42 | 2 | 25 | 7 | 42 |
| Late-career (Prof and Heads of dept) | 1 | 10 | 0 | 0 | 1 | 13 | 1 | 6 |
| Missing | 0 | 0 | 0 | 0 | 0 | 0 | 0 | 0 |
| ***Croatia*** |  |  |  |  |  |  |  |  |
| PhD student | 1 | 7 | 1 | 8 | 1 | 17 | 1 | 6 |
| Mid-career (Post doc, senior researcher, assit. or assoc. prof) | 5 | 34 | 6 | 47 | 3 | 50 | 6 | 36 |
| Late-career (Prof and Heads of dept) | 4 | 27 | 2 | 16 | 2 | 34 | 4 | 24 |
| Missing | 1 | 7 | 0 | 0 | 0 | 0 | 1 | 6 |
| ***All*** |  |  |  |  |  |  |  |  |
| PhD student | 3 | 8 | 7 | 19 | 6 | 27 | 9 | 16 |
| Mid-career (Post doc, senior researcher, assit. or assoc. prof) | 14 | 35 | 14 | 37 | 9 | 40 | 20 | 34 |
| Late-career (Prof and Heads of dept) | 7 | 18 | 3 | 8 | 4 | 18 | 8 | 14 |
| Missing | 1 | 3 | 2 | 6 | 0 | 0 | 3 | 6 |

^a^ The representation of different stakeholder groups over all participants (n=59) is not the sum of rounds 1 to 3 because many stakeholders participated in multiple rounds.

*Number of participants who identified as researchers who did not indicate a departmental position
